# Hepatoprotective Effects of Quercetin‐Chitosan Nanoparticles on Nonalcoholic Fatty Liver Disease: In Vivo Study

**DOI:** 10.1002/fsn3.71617

**Published:** 2026-04-08

**Authors:** Behina Babaalizadeh, Mahmoud Osanloo, Alireza Tavassoli, Elham Zarenezhad, Sedigheh Niknia, Behnoosh Miladpour

**Affiliations:** ^1^ Student Research Committee Fasa University of Medical Sciences Fasa Iran; ^2^ Department of Medical Nanotechnology, School of Medicine Fasa University of Medical Sciences Fasa Iran; ^3^ Department of Pathology, School of Medicine Fasa University of Medical Sciences Fasa Iran; ^4^ Noncommunicable Diseases Research Center Fasa University of Medical Sciences Fasa Iran; ^5^ Department of Clinical Biochemistry, School of Medicine Fasa University of Medical Sciences Fasa Iran

**Keywords:** chitosan nanoparticle, fatty liver, NAFLD, nanoparticle, nonalcoholic fatty liver disease, quercetin

## Abstract

Metabolic dysfunction associated steatotic liver disease (MASLD) is a common chronic liver disorder characterized by hepatic steatosis, inflammation, and progression to fibrosis and cirrhosis. Despite advances in pharmacotherapy, including thyroid hormone receptor β agonists (resmetirom) and GLP 1 receptor agonists, no universally accepted curative treatment exists. Nutraceuticals such as quercetin offer promising hepatoprotective effects, yet their clinical translation is limited by low bioavailability and stability. In this study, we evaluated the therapeutic potential of chitosan‐encapsulated quercetin (Que‐CH) nanoparticles in a high‐fat diet rat model of MASLD. Que‐CH nanoparticles were characterized by particle size (~190 nm), positive zeta potential (+56.5 mV), and 65% encapsulation efficiency. Treatment with Que‐CH significantly reduced serum IL‐1β, improved liver histology by decreasing macro‐ and microvesicular steatosis, and lowered the Bax/Bcl‐2 ratio, indicating reduced hepatocyte apoptosis. These results suggest that Que‐CH nanoparticles enhance the bioactivity of quercetin, exerting anti‐inflammatory, anti‐apoptotic, and lipid‐lowering effects in MASLD. This study highlights the potential of nanocarrier‐based nutraceutical interventions as complementary strategies for liver steatosis management, while further mechanistic studies and pharmacokinetic evaluations are warranted to support clinical translation.

## Graphical abstract

Quercetin‐chitosan Nanoparticles ameliorated hepatic steatosis, inflammation, and dyslipidemia, leading to significant improvement of liver injury in a rat model of Nonalcoholic Fatty Liver Disease.

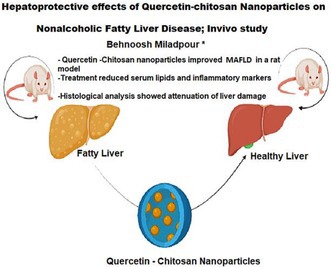

## 1 Introduction

Nonalcoholic fatty liver disease (NAFLD), recently redefined as metabolic dysfunction associated steatotic liver disease (MASLD), is among the most prevalent chronic liver disorders, affecting approximately 25% of the global population (Xu et al. 2019). Its pathogenesis involves lipid peroxidation, oxidative stress, inflammation, and insulin resistance. MASLD encompasses a spectrum of liver conditions, ranging from simple steatosis to steatohepatitis, fibrosis, and cirrhosis (Zhou et al. 2022; Durand et al. 2021; Barbier‐Torres et al. 2020; Kamata et al. 2005). Mitochondrial dysfunction and excess reactive oxygen species (ROS) contribute to hepatocyte damage, activating inflammatory pathways such as nuclear factor kappa B (NF κB) and promoting secretion of pro‐inflammatory mediators including interleukin (IL)‐1β, IL‐6, tumor necrosis factor (TNF)‐α, CCL2, and CXCL1 (Mascaró et al. 2022; Rau and Geier 2021; Valencia et al. 2021). Despite increasing prevalence, no universally approved pharmacological treatment exists. Recent clinical advances, however, have introduced promising agents: the thyroid hormone receptor β agonist resmetirom has shown reductions in hepatic fat content, improvements in liver enzymes, and favorable histological outcomes, but it is not yet considered a definitive cure for MASLD, as long‐term efficacy and safety remain under investigation (Suvarna et al. 2024). Similarly, GLP 1 receptor agonists have demonstrated potential in reducing liver fat and improving metabolic parameters, yet their use is largely supportive and requires further validation in larger clinical trials (Njei et al. 2024). Given these limitations, dietary interventions and bioactive compounds remain of interest. Quercetin, a naturally abundant flavonoid found in vegetables, fruits, and red wine, has been reported to possess antioxidant, anti‐inflammatory, and hepatoprotective properties (Rashedi et al. 2019; Zhao et al. 2022). However, its poor water solubility and low bioavailability limit its therapeutic potential. Nanoparticle‐based delivery systems, such as chitosan‐encapsulated quercetin (Que‐CH), may overcome these barriers by enhancing solubility, stability, and cellular uptake, thereby improving its efficacy in experimental models of liver disease (Patel et al. 2024; Roy et al. 2020, 2022). Therefore, we hypothesized that nanoencapsulation of quercetin within a chitosan matrix would enhance its bioavailability and consequently exert superior hepatoprotective effects compared with free quercetin by simultaneously attenuating steatosis, inflammation, and hepatocyte apoptosis. The novelty of this study lies in the development and in vivo evaluation of a quercetin‐loaded chitosan nanoformulation as a targeted nano‐nutraceutical approach for MASLD, integrating nanotechnology with dietary bioactive therapy to overcome the pharmacokinetic limitations of conventional flavonoid supplementation. To comprehensively assess therapeutic efficacy, we selected clinically and mechanistically relevant endpoints, including anthropometric and liver indices (metabolic burden), histopathological steatosis scoring (lipid accumulation), inflammatory cytokines (immune activation), apoptotic markers (mitochondrial injury), and lipid profiles (metabolic dysregulation). Together, these endpoints enable multidimensional evaluation of disease progression and treatment response. Accordingly, this study aimed to determine whether Que‐CH nanoparticles could provide a multi‐target, translational strategy for the management of MASLD in a high‐fat diet rat model.

## 2 Material and Methods

This study was conducted during 2022–2023 at Fasa University of Medical Sciences. The research committee approved all experimental procedures, with the grant code: 400286. Chemicals used in this study were purchased from Sigma‐Aldrich (USA), including HPLC 95% grade quercetin (Que), low molecular weight chitosan (CH; 448869), and sodium tripolyphosphate (STPP; 72061). Additional chemicals, including cholesterol (C8667), saccharose (1‐50‐57), SDS (106022), glycine (56‐40‐6), methanol (67‐56‐1), and Tris base (77‐86‐1), were acquired from Merck, Germany. The Pierce BCA protein assay kit (23225) was obtained from Thermo Fisher, USA. Anti‐Bax (ab32503) and anti‐Bcl‐2 (Abcam 59348) antibodies were sourced from Abcam, UK, and a secondary antibody (HRP monoclonal; 405306) was purchased from BioLegend, USA.

### 2.1 Que‐CH Nanoparticle Synthesis

Chitosan (CH) powder was initially dissolved overnight at room temperature in 1% acetic acid with magnetic stirring at 2000 rpm. Then, quercetin (Que) (5% w/v) was dissolved in 96% alcohol, concentrating at 0.5% v/v. Tween 80 was added and mixed for 5 min. The chitosan solution was then gradually dripped into the mixture. Sodium tripolyphosphate (TPP) at 0.1% was previously dissolved in distilled water and was added dropwise to the mixture, which was then stirred at 2000 rpm for 40 min to ensure homogenization. The resulting nanoparticle suspension was adjusted to pH 7.0 and used directly for subsequent in vivo administration without further isolation.

#### 2.1.1 Nanoparticle Characteristics

Dynamic light scattering (DLS) at a 90° angle and a temperature of 25°C (SZ100, Horiba, USA) was used to determine the particle size, size distribution, and zeta potential (surface charge). The morphology of the nanoparticles dispersed in the chitosan solution was further confirmed using transmission electron microscopy (TEM).

#### 2.1.2 ATR‐FTRI (Fourier Transform Infrared)

To analyze the characteristics of the nanoparticles, Fourier‐transform infrared (FTIR) spectroscopy was performed using a TENSOR II instrument on the blank nanoparticle, quercetin (Que), and Que‐CH nanoparticle solutions. The analysis was carried out using the KBr disk method in the scanning range of 400–4000 cm^−1^ with a resolution of 2 cm^−1^.

#### 2.1.3 Investigation of Encapsulation Efficiency

Chitosan nanoparticles containing quercetin were prepared using the ionic gelation method. Following nanoparticle formation, the suspension was centrifuged at 20,000 × g for 30 min at 4°C to ensure complete sedimentation of the nanoparticles. The supernatant was separated, and the amount of free quercetin in it was determined using a quercetin calibration curve constructed at a wavelength of 425 nm. To construct the calibration curve, a stock solution of quercetin with a concentration of 1000 μg/mL was prepared in absolute ethanol. Serial dilutions were then performed to obtain concentrations ranging from 1.56 to 12.50 μg/mL, ensuring absorbance values remained within the linear range of the spectrophotometer (below 2). The linear regression equation derived from the calibration curve was *y* = 0.1309*x* + 0.0273 with an *R* ^2^ value of 0.9994. This equation was used to calculate the quercetin concentration in the supernatant. Encapsulation efficiency of the nanoparticles was calculated using the following formula:

$$\begin{array}{c} \text{Encapsulation efficiency}\;\left(\%\right)=\\ \text{Initial amount of quercetin}–\text{Free quercetin in supernatant}/\\ \text{Initial amount of quercetin}\times 100. \end{array}$$

#### 2.1.4 DPPH Assay (2, 2‐Diphenyl‐1‐Picrylhydrazyl)

The DPPH assay was used to assess the antioxidant effect of the nanoparticles (Que dissolved in water or DMSO, along with its nanoparticle formulation). For this, 125 μL of each sample was combined with 125 μL of DPPH‐ethanol solution and incubated at room temperature. The DPPH compound in the ethanol solution was reduced to DPPH_2_ after 30 min due to the antioxidant activity of the Que‐CH nanoparticles. The absorbance at 517 nm was measured using a spectrophotometer.

### 2.2 Animals and Inducing Nonalcoholic Fatty Liver

Twenty‐four male Wistar rats (4–5 weeks old, weighing 180 ± 20 g) were randomly selected and housed in four cages to acclimate to standard laboratory conditions (temperature of 25°C ± 2°C, relative humidity of 50%–70%, and a 12‐h light/dark cycle). After a 1‐week adaptation period, they were randomly divided into four groups (6 rats per group): (1) Control (C), (2) Control + therapy with Que‐CH (control + Que nano), (3) MASLD (receiving a high‐fat diet in addition to their standard chow diet, fatty liver), and (4) MASLD + treatment with Que‐CH nanoparticles (fatty liver + Que nano). To induce fatty liver, Yuhong Zou's method was used (Zou et al. 2006). A high‐fat emulsion was prepared and administered orally at a dose of 1 mg/kg per day for 10 weeks, in addition to their regular diet and water ad libitum. This high‐fat emulsion contained 77% of its total energy from fat, 14% from whole milk powder, and 9% from carbohydrates. The composition of the emulsion is shown in Table 1.

**TABLE 1:** Composition of high‐fat emulsion and yielded energy.

Component	High‐fat emulsion
Corn oil	400 g
Saccharose	150 g
Milk powder	80 g
Cholesterol	100 g
Tween 80	36.4 g
Table salt	10 g
Distilled water	300 mL
Total energy	4342 kcal/L

This research was approved by the research center's committee under the ethical code IR.FUMS.AEC.1401.002. As per previous studies, the fatty liver group was sacrificed under anesthesia with ketamine‐xylazine after 12 h of fasting and 10 weeks of receiving a high‐fat diet. Plasma was collected at −20°C for further analysis. Additionally, the liver was weighed, rinsed with 0.9% normal saline, and each section was either frozen at −80°C or fixed with 10% formalin. Following the successful induction of MASLD, the high‐fat diet was discontinued. In line with prior research, Que‐CH nanoparticle treatment was administered daily for 30 days at a dose of 30 mg/kg and continued for an additional 30 days. Similarly, in the positive control group (control + Que nano), Que‐CH nanoparticles were administered daily without disease induction for 30 days at 30 mg/kg by gavage. After this treatment, the animals were weighed and sacrificed. Plasma and liver samples were collected, weighed, and rinsed with 0.9% normal saline.

#### 2.2.1 Lipid Profile (LFT) and Proinflammatory Cytokine (IL‐1β)

Liver function tests (LFTs), including serum glutamic oxaloacetic transaminase (SGOT, AST), serum glutamic pyruvic transaminase (SGPT, ALT), gamma‐glutamyl transferase (GGT), alkaline phosphatase (ALK), total protein (TP), albumin (Alb), and total bilirubin (TB), were measured to assess the extent of liver damage. Additionally, lipid profiles, including triglycerides (TG), cholesterol (Chol), HDL, and LDL, were evaluated to determine liver lipid content. All tests were performed using the Biorex Fars kit.

Interleukin‐1β (IL‐1β), a proinflammatory cytokine, was measured using the enzyme‐linked immunosorbent assay (ELISA) method with the Karmania Pars Gene kit.

#### 2.2.2 Histopathological Examination

Hematoxylin–eosin (H&E)‐stained formalin‐fixed liver tissue was used to assess liver pathological status, including lipid content, inflammation, and apoptotic factors. Fresh frozen tissue was utilized for western blot analysis.

#### 2.2.3 Western Blot

Apoptotic factors were evaluated using Western blotting before and after treatment. To extract liver protein, frozen liver sections were homogenized with RIPA buffer to lyse hepatocytes, and a protease inhibitor was added to inhibit proteases in the solution. Total protein (30 μL of lysate) was loaded onto a 12.5% SDS‐PAGE gel and run at 120 V for two hours. The protein was then transferred to a nitrocellulose membrane, blocked with 5% skim milk, and incubated overnight at 4°C. Following this, a second overnight incubation at 4°C was performed with the primary antibody (anti‐Bax, anti‐Bcl‐2, Abcam, 1:2000 dilution). After washing the membrane with TBS‐T buffer, a secondary antibody (horseradish peroxidase (HRP), Abcam, 1:5000 dilution) was added and incubated for one hour at 4°C. The bond was detected by chemiluminescence (ECL). Finally, optical density was analyzed using ImageJ software.

### 2.3 Statistical Analysis

Statistical analysis was performed using SPSS (v. 25, IBM Corp., USA). One‐way analysis of variance (ANOVA) followed by Tukey's post hoc test was used to compare the data. A *p*‐value ≤ 0.05 was considered statistically significant.

## 3 Results

### 3.1 Nanoparticle Characterizations

#### 3.1.1 Characterization of Que Encapsulated With CH

**FIGURE 1:**
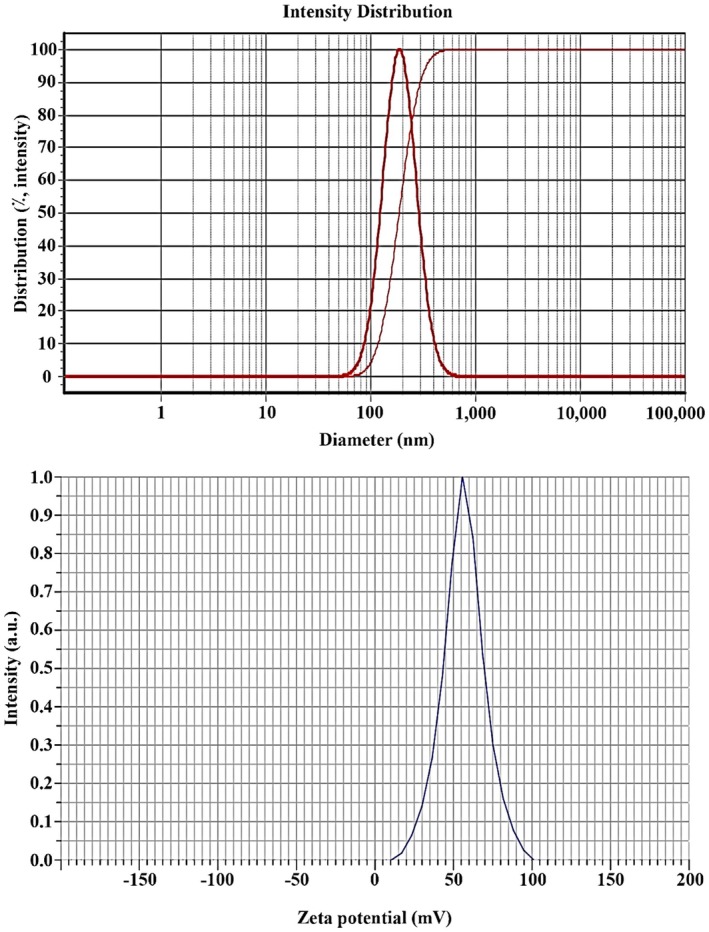
DLS nanoparticle size distribution (top); the mean diameter was 190 nm with a span of 0.94 nm, and the nanoparticle's surface charge was +56.5 mV (below).

**FIGURE 2:**
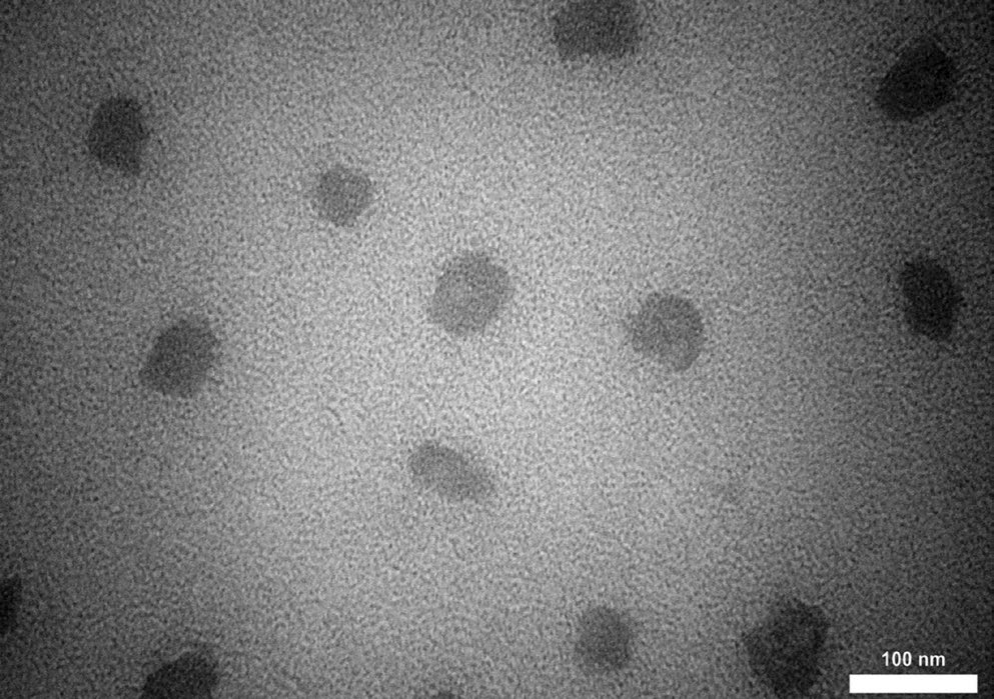
TEM image showing the round shape of Que encapsulated with CH and TPP.

The synthesized Que‐CH nanoparticles exhibited a mean diameter of 190 nm with a narrow size distribution (span of 0.94), as determined by DLS (Figure 1). Crucially, the zeta potential was measured to be strongly positive at +56.5 mV (Figure 1). TEM imaging confirmed the spherical morphology of the nanoparticles and showed quercetin encapsulated within a porous chitosan matrix (Figure 2). These physicochemical characteristics—a sub‐200 nm size and a high positive surface charge—are critically important for the nanoparticle's stability and biological interactions. A summary of all data is shown in Table 2. and Figure 1.

**TABLE 2:** Examining the optimal concentration for fabricating Que‐CH nanoparticles.

Sample number	Chitosan (%)	TPP^a^	Tween 20 (%)	Tween 80 (%)	Mixed Tween	Ethanol (%)	Que^b^	Span	Particle size (nm)
1	0.55	0.1	7			0.5	25	0.94	807
2	0.55	0.03	7			0.5	25	0.95	653
3	0.55	0.05	7			0.5	25	0.94	568
4	0.55	0.2	7			0.5	25	0.95	710
5	0.55	0.1		7		0.5	25	0.97	249
6	0.55	0.03		7		0.5	25	0.94	223
7	0.55	0.05		7		0.5	25	0.96	958
8	0.55	0.2		7		0.5	25	0.96	562
9	0.55	0.1			3.5 + 3.5	0.5	25	0.97	392
10	0.55	0.03			3.5 + 3.5	0.5	25	0.97	504
11	0.55	0.05			3.5 + 3.5	0.5	25	0.94	398
12	0.55	0.2			3.5 + 3.5	0.5	25	0.97	607
13	0.55	0.1		7		0.5	25	0.94	190

^a^ Sodium triple phosphate.

^b^ Quercetin.

#### 3.1.2 ATR‐FTIR

**FIGURE 3:**
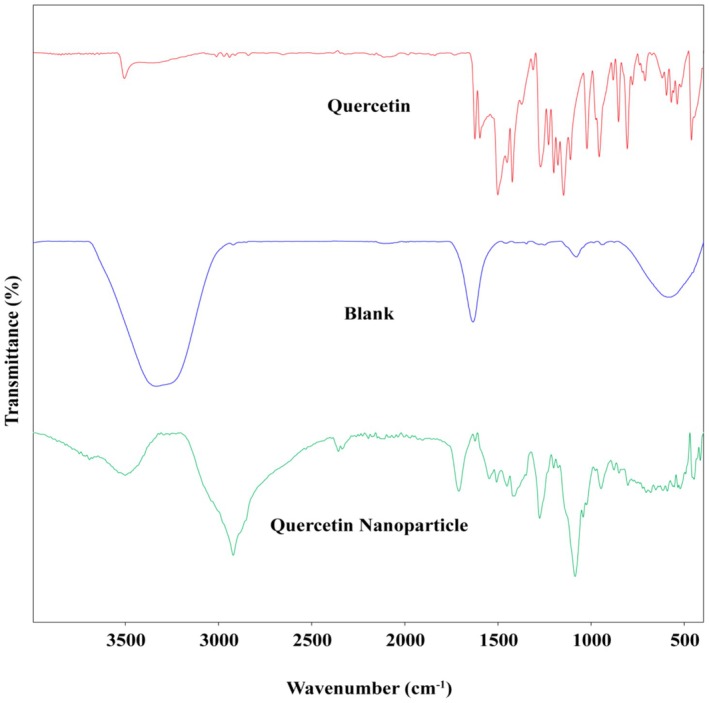
ATR‐FTIR spectra of blank, Que, and Que‐CH nanoparticles.

Attenuated total reflection (ATR)‐FTIR spectra of quercetin (Que) showed broad and characteristic bands at 3508 cm^−1^, corresponding to the OH stretching vibration in phenolic hydroxyl bonding. The bands at 3014 cm^−1^ are attributed to =C–H stretching, while the characteristic bands at 2974, 2944, and 2843 cm^−1^ represent the stretching vibrations of –CH groups due to sp^3^ hybridization. The band at 1734 cm^−1^ is attributed to the carbonyl group, and the bands at 1627 and 1454 cm^−1^ correspond to C=C vibrations. The band at 1376 cm^−1^ is associated with the aromatic ring of the phenolic moiety in Que, while the band at 1314 cm^−1^ corresponds to =C–O–H stretching, and the peak at approximately 1151 cm^−1^ is linked to C–O bending vibrations. The band at 885 cm^−1^ is related to the vibrational stretching of the catechol moiety. The ATR‐FTIR blank spectra exhibited broad bands between 3200 and 3600 cm^−1^, corresponding to the OH stretching vibration resulting from hydrogen bonding in chitosan (CH), sodium tripolyphosphate (TPP), and Tween. The band at 2924 cm^−1^ is attributed to the C‐H bond, while the band at 1637 cm^−1^ corresponds to a carbonyl group. The characteristic band at 945 cm^−1^ is related to symmetric and anti‐symmetric stretching vibrations in the PO_2_ group, while the prominent band at 1082 cm^−1^ is associated with symmetric and anti‐symmetric stretching vibrations in the PO_3_ group. The FTIR spectra of the Que‐CH nanoparticles revealed a broad band between 3300 and 3750 cm^−1^, corresponding to the OH stretching vibration due to hydrogen bonding in CH, TPP, and Que. The stretching at 2924 cm^−1^ is associated with C‐H bonds in both CH and Que. Peaks at 2359 and 2339 cm^−1^ correspond to CO_2_ vibrations, while the band at 1279 cm^−1^ is attributed to C–N stretching. This suggests the formation of a complex due to electrostatic interactions between the NH_3_^+^ groups of CH and the phosphoric groups of TPP within the nanoparticles. TPP ions may have contributed to increased ionic crosslinking between the ‐NH_3_^+^ groups of CH. Additional bands for Que and the nanoparticles confirmed that Que was successfully loaded into the nanoformulation. The FTIR spectra are depicted in Figure 3.

#### 3.1.3 DPPH

The DPPH test demonstrated an 83% antioxidant effect at a wavelength of 517 nm, with a mean value of 83.401 ± 0.856.

#### 3.1.4 Quercetin Calibration Curve and Its Encapsulation Efficiency

**FIGURE 4:**
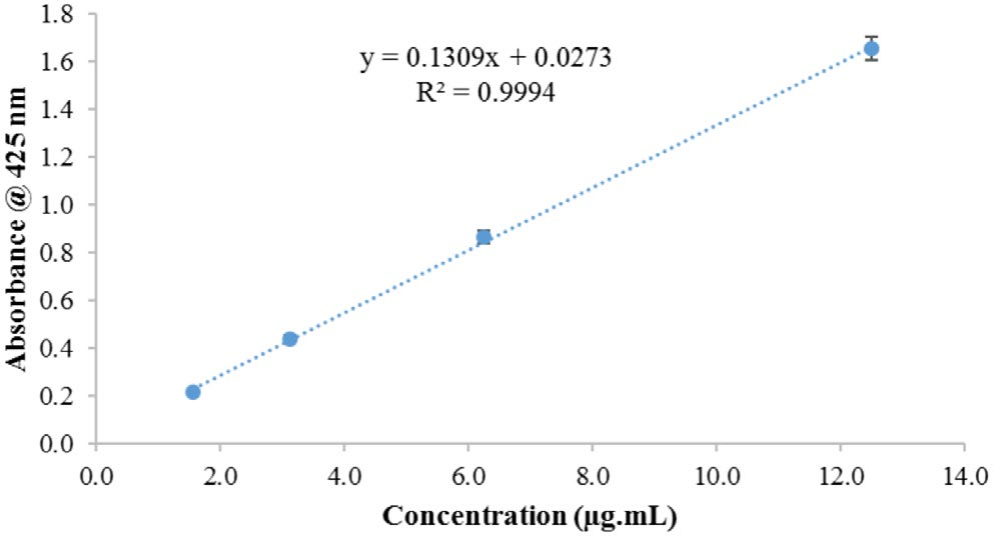
Quercetin calibration curve.

Based on the plotted calibration curve, the encapsulation efficiency of the nanoparticles was determined to be 65%. These results highlight the significant efficacy of the ionic gelation method in loading quercetin into chitosan nanoparticles, Figure 4.

Chitosan nanoparticles containing quercetin were prepared using the ionic gelation method. Following nanoparticle formation, the suspension was centrifuged at 20,000 × ***g*** for 30 min at 4°C to ensure complete sedimentation of the nanoparticles. The supernatant was separated, and the amount of free quercetin in it was determined using a quercetin calibration curve constructed at a wavelength of 425 nm. To construct the calibration curve, a stock solution of quercetin with a concentration of 1000 μg/mL was prepared in absolute ethanol. Serial dilutions were then performed to obtain concentrations ranging from 1.56 to 12.50 μg/mL, ensuring absorbance values remained within the linear range of the spectrophotometer (below 2). The linear regression equation derived from the calibration curve was *y* = 0.1309*x* + 0.0273 with an *R* ^2^ value of 0.9994. This equation was used to calculate the quercetin concentration in the supernatant. Encapsulation efficiency of the nanoparticles was calculated using the following formula:

$$\begin{array}{c} \text{Encapsulation efficiency}\;\left(\%\right)=\\ \text{Initial amount of quercetin}–\text{Free quercetin in supernatant}/\\ \text{Initial amount of quercetin}\times 100 \end{array}$$

Based on this, the encapsulation efficiency of the nanoparticles was determined to be 65%. These results highlight the significant efficacy of the ionic gelation method in loading quercetin into chitosan nanoparticles.

### 3.2 Proved NAFLD and Treatment With Que‐CH Nanoparticle

#### 3.2.1 The Effect of Que‐CH Nanoparticles on Body Weight, Liver Weight, Liver Density, and Liver/Body Ratio

**FIGURE 5:**
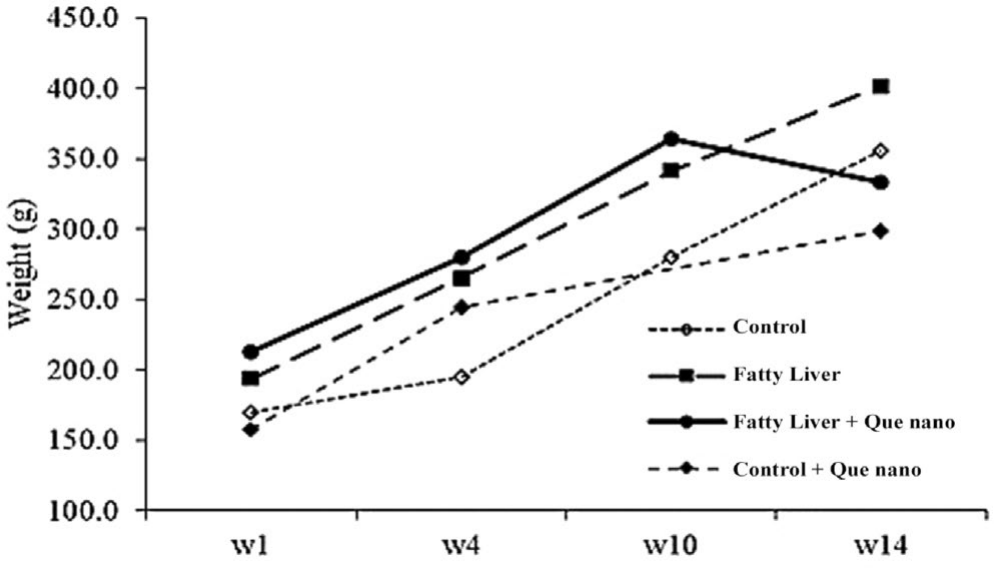
Body weight in groups in Weeks 1, 4, 10, and 14. The results showed a significant decrease at Week 14 (*p*‐value = 0.002).

This study involved 24 male Wistar rats (4–5 weeks old, weighing 180 ± 20 g). The rats were randomly divided into four groups, each consisting of six rats: (1) Control (C), (2) Control + therapy with Que‐CH (control + Que nano), (3) NAFLD, and (4) NAFLD + treatment utilizing Que‐CH nanoparticles (fatty liver + Que nano). Fatty liver was induced using a high‐fat emulsion (1 mg/kg/day) for 10 weeks. Afterward, the control nano‐ and fatty liver groups were treated with Que nano for 30 days. Following the treatment period, all groups were sacrificed, and their weight, liver weight, liver density, and liver/body ratio were calculated. The results of the weights are shown in Figure 5 and Table 3. A statistically significant difference was observed between the fatty liver and fatty liver + Que nano groups at Week 14 *p*‐value = 0.002.

**TABLE 3:** The liver weight, liver density, and liver‐to‐body ratio between *p* value ≤ 0.05.

Groups parameters	Control	Control + Que nano	*p*	Fatty liver	Fatty liver + Que nano	*p*
Liver weight (g)	10.67 ± 3.27	9.50 ± 1.91	0.089	13.2 ± 2.17	10.75 ± 1.50	0.046*
Liver density	1.76 ± 0.6	1.57 ± 0.46	0.889	1.04 ± 0.28	1.13 ± 0.17	0.99
Liver weight/body weight ratio	0.028 ± 0.008	0.026 ± 0.005	0.97	0.035 ± 0.008	0.028 ± 0.005	0.39

#### 3.2.2 The Effect of Que‐CH Nanoparticles on Serum Parameters (LFT/Lipid Profile and IL‐1β)

**FIGURE 6:**
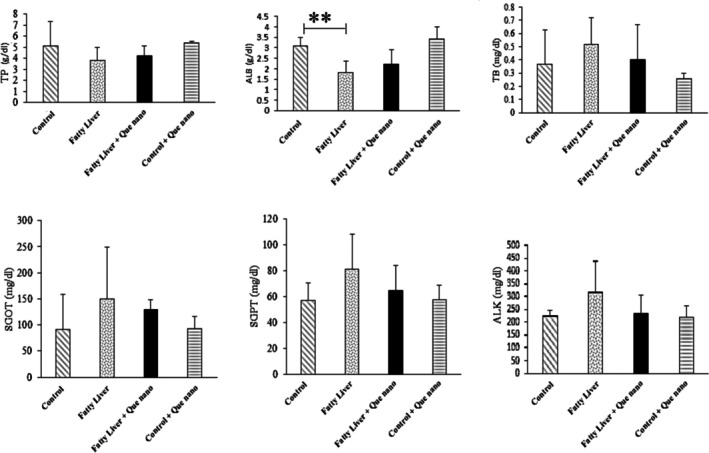
Comparing LFT tests between the study groups. Albumin was significantly lower in the fatty liver group than in the control group; *p*‐values = 0.004. No significant difference was seen in other parameters between groups. **p‐value ≤ 0.01.

**FIGURE 7:**
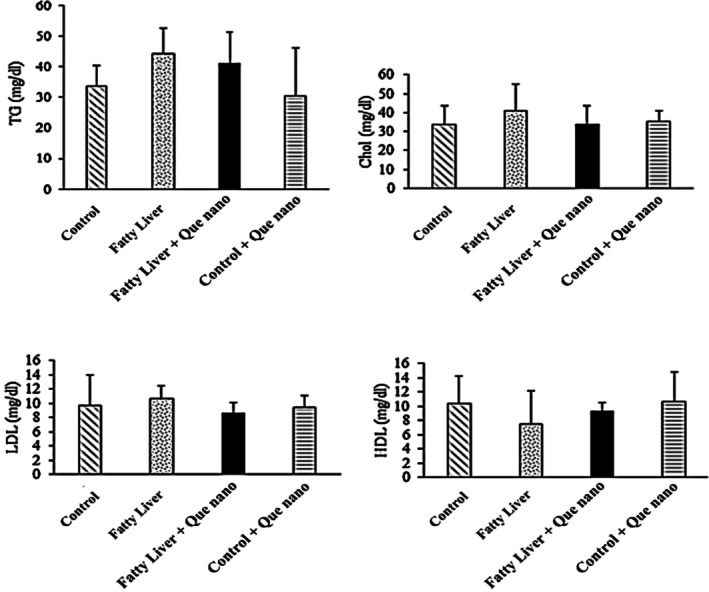
Comparison of the lipid profiles: TG, Chol, HDL, and LDL between study groups. None are significant.

**FIGURE 8:**
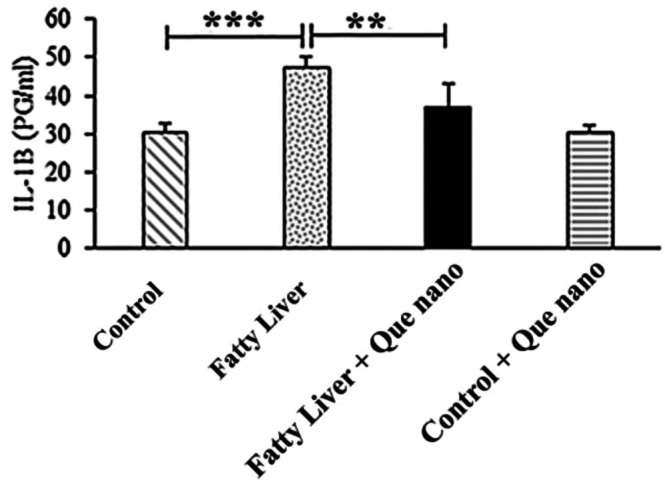
Serum levels of IL‐1β in the study groups. A significantly higher level of IL‐1β was found in the fatty liver group than in the control group (*p* value < 0.001). Additionally, we demonstrated a significant reduction in the serum level of IL‐1β in the fatty liver + Que nano group compared to the fatty liver group (*p* value = 0.009). **p‐value ≤ 0.01; ***p‐value ≤ 0.001.

The serum LFT/lipid profile results and IL‐1β levels are shown in Figures 6, 7, 8. There were no significant differences between groups in the LFT/lipid profile parameters, except for serum albumin (Alb), which was lower in the fatty liver group compared to the control group (*p*‐value = 0.004; Figure 6). Additionally, IL‐1β levels were significantly higher in the fatty liver group than in the control group (*p*‐value = 0.001). After treatment with Que nanoparticles, IL‐1β levels were dramatically reduced in the fatty liver + Que nano group (*p*‐value = 0.009; Figure 8).

#### 3.2.3 Liver Histopathology Changes

**FIGURE 9:**
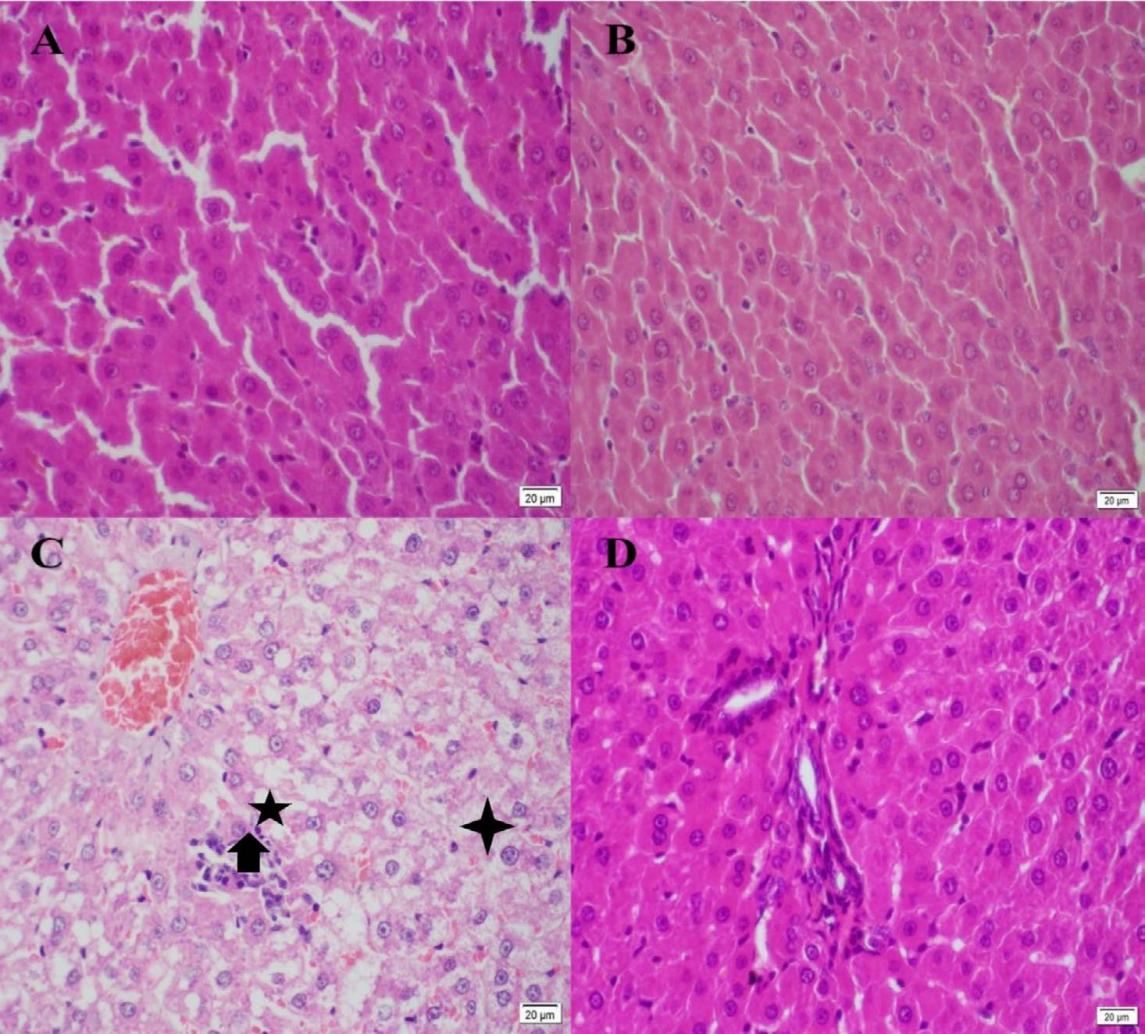
Histopathology results. H&E staining, ×400, (A) Control groups, (B) Control + quercetin nanoparticle, (C) Fatty liver, (D) Fatty liver+ quercetin nanoparticle, microvesicular/macrovesicular lipid droplets are evident in the fatty liver group that are marked in the figure. After administration of the quercetin nanoparticles, lipid droplets were diminished in the fatty liver+ quercetin nanoparticle group. 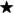 Macrovesicular lipid droplet. 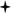 Microvesicular lipid droplet. 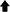 Inflammation center and apoptotic body.

The present study identified three factors as signs of steatosis in the liver: ballooning degeneration, inflammation, and microvesicular/macrovesicular lipid droplets. These criteria confirm our biochemical analysis. According to the grading system developed by Kleiner et al. (Kleiner et al. 2005) and pathologist verification, NAFLD Grade 3 was successfully induced. In a microscopic liver section, lobules contain three zones: zone 1 (periportal triad zone; a combination of the portal vein, hepatic artery branch, and bile duct), zone 2 (mid‐zone), and zone 3 (precentral vein). Macro/microvesicular lipid droplets were observed in the fatty liver section. In addition, granulomatous or inflammatory centers (lymphocyte infiltration) and apoptosis were present. The majority of steatosis was observed in zones 2 and 3. After treatment with Que nanoparticles, steatosis was eliminated. Furthermore, inflammatory centers and apoptosis were significantly reduced in all zones. The pathological outcome is depicted in Figure 9.

#### 3.2.4 Western Blot

**FIGURE 10:**
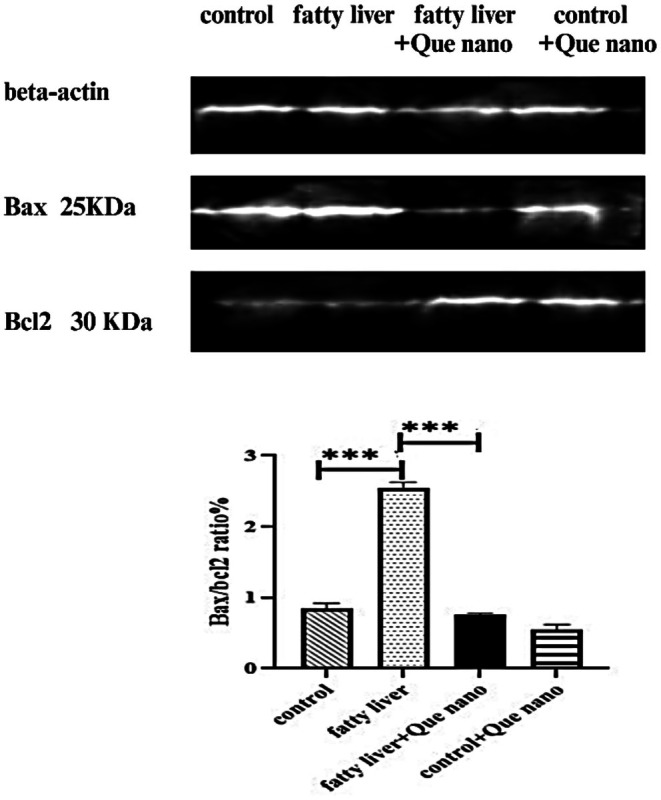
Western blot in comparing the Bax/BCL2 ratio between study groups, the BAX/BCL2 ratio was significantly reduced after quercetin nanoparticle administration in the fatty liver + Que nano group (*p*‐value ≤ 0.001), significantly increased after Que nanoparticle administration (*p*‐value ≤ 0.001), and no significant difference in the Bax/BCL2 ratio was found in the control + Que nano group compared with the control group. ****p‐value ≤ 0.001.

After the administration of Que nanoparticles, the levels of Bax/Bcl‐2 were lower in the Que‐CH nanoparticle group compared to the fatty liver group. Scanning with ImageJ software revealed a significant difference between the groups treated with Que‐CH nanoparticles (fatty liver + Que nano) and the fatty liver group (*p*‐value ≤ 0.001). Additionally, there was a significant increase in apoptosis in the fatty liver group compared to the control group (*p*‐value ≤ 0.001). No significant difference was observed between the control and control + Que nano groups, as shown in Figure 10.

## 4 Discussion

The present study investigated the therapeutic efficacy of quercetin‐loaded chitosan nanoparticles (Que‐CH) as a targeted nano‐nutraceutical strategy. Our findings demonstrate that Que‐CH administration significantly improved anthropometric indices (body and liver weight), liver density, histopathological steatosis scores, inflammatory cytokines, and apoptotic markers. Importantly, these improvements were not merely descriptive but reflect coordinated modulation of multiple pathogenic axes of MASLD, including lipid metabolism, oxidative stress, inflammation, and programmed cell death. This multi‐target activity is particularly valuable, as MASLD pathogenesis is driven by interconnected mechanisms rather than a single pathway.

### 4.1 Modulation of Hepatic Steatosis and Lipid Metabolism

Hepatic steatosis represents the earliest and central event in MASLD progression. In the current study, Que‐CH markedly reversed macrovesicular steatosis and histological abnormalities, indicating improved lipid handling in hepatocytes. These observations align with previous mechanistic studies demonstrating that quercetin suppresses de novo lipogenesis and enhances fatty acid oxidation. Gnoni et al. reported that quercetin limits lipid accumulation via regulation of the AMPK/PP2A/ACC1 signaling axis (Gnoni et al. 2022), whereas Son et al. showed reduced cholesterol and triglyceride deposition following quercetin nanoemulsion treatment (Son et al. 2019). Consistent with these findings, our data suggest that the nanoformulated quercetin enhances metabolic reprogramming toward lipid utilization rather than storage. From a translational perspective, such lipid‐lowering effects are highly relevant because excessive triglyceride deposition is strongly associated with mitochondrial dysfunction and disease progression (Durand et al. 2021; Barbier‐Torres et al. 2020). Therefore, the reduction in steatosis observed here likely represents an upstream event contributing to the overall hepatoprotective outcome.

### 4.2 Anti‐Apoptotic Effects and Hepatocyte Survival

Apoptosis is a key driver of liver injury and inflammation in MASLD. We observed a significant reduction in the Bax/Bcl‐2 ratio following Que‐CH treatment, indicating suppression of mitochondrial‐mediated apoptosis. This effect corroborates earlier evidence that quercetin modulates apoptotic regulators and preserves mitochondrial integrity (Li et al. 2021). Given that reactive oxygen species activate JNK‐dependent apoptotic pathways in steatotic hepatocytes (Kamata et al. 2005), the decreased apoptotic signaling observed here likely reflects attenuation of oxidative stress–induced mitochondrial damage. Thus, Que‐CH appears to promote hepatocyte survival not only by limiting lipid overload but also by stabilizing mitochondrial homeostasis, providing a dual protective mechanism.

### 4.3 Anti‐Inflammatory Activity

Chronic low‐grade inflammation is a hallmark of MASLD progression from steatosis to steatohepatitis. Our study demonstrated a marked reduction in IL‐1β levels in Que‐CH–treated animals, supporting potent anti‐inflammatory activity. Quercetin has consistently been shown to inhibit pro‐inflammatory cytokines such as IL‐1β, IL‐6, and TNF‐α (Wu et al. 2020), and nanoencapsulation further amplifies this effect by improving cellular delivery (Choudhary et al. 2020). Considering that cytokine‐driven inflammation exacerbates hepatocyte injury and fibrosis, the suppression of inflammatory mediators observed here likely contributes significantly to the histological improvements. These findings emphasize that Que‐CH acts not simply as an antioxidant supplement but as an immunometabolic modulator.

#### 4.3.1 Antioxidant Properties

The utilization of chitosan as a nanocarrier for quercetin represents a strategic approach to overcome the inherent limitations of free quercetin in NAFLD management. The formation of stable Que‐CH nanoparticles, confirmed in our study, is primarily attributed to the ionic gelation mechanism between the positively charged amino groups of chitosan and the negatively charged phosphate groups of TPP. This cross‐linking creates a dense, biocompatible matrix that efficiently encapsulates the hydrophobic quercetin molecules. The high positive zeta potential of +56.5 mV confirmed the excellent colloidal stability of the formulation, preventing aggregation and ensuring consistent performance during the study. This nanoformulation directly addresses the poor aqueous solubility and low bioavailability of quercetin, not only by enhancing its dissolution but also by protecting it from premature degradation and facilitating its absorption. Furthermore, this strong positive surface charge promotes mucoadhesion and interaction with negatively charged cell membranes, which enhances cellular uptake in the liver. Our decision to administer the freshly prepared nanoparticle suspension, without a drying step, ensured that the nanoparticles were delivered in their native, well‐dispersed state, maximizing their interaction with the biological environment. The significant hepatoprotective effects observed—including reduced inflammation, apoptosis, and steatosis—are therefore not solely a consequence of quercetin's bioactivity but are likely amplified by chitosan's role as an effective nanocarrier. This synergy between the drug and its delivery system underscores the potential of Que‐CH nanoparticles as a promising therapeutic strategy for NAFLD (Patel et al. 2024; Roy et al. 2022; Li et al. 2021; AlNafea and Korish 2021; Lu et al. 2021; Essa et al. 2022).

#### 4.3.2 Chitosan as a Nanocarrier

The use of chitosan as a carrier for quercetin nanoparticles represents a novel approach for the treatment of NAFLD. Chitosan not only enhances the solubility and bioavailability of quercetin, which is otherwise poorly water‐soluble, but also serves as a biodegradable polymer that facilitates targeted drug delivery to the liver. Our findings are consistent with previous studies demonstrating the hepatoprotective effects of chitosan‐based nanoparticles in drug delivery systems (Li et al. 2021; Lu et al. 2021; Zou et al. 2006; Kleiner et al. 2005). The combination of quercetin's bioactive properties and chitosan's nanocarrier capabilities suggests that Que‐CH nanoparticles may represent a promising therapeutic strategy for NAFLD.

### 4.4 Clinical and Translational Context

Although lifestyle modification remains first‐line therapy (Mascaró et al. 2022), pharmacological options for MASLD are still evolving. Recently, thyroid hormone receptor‐β agonists such as resmetirom and GLP‐1 receptor agonists have shown promising clinical benefits in reducing steatosis and fibrosis, highlighting the need for multi‐target approaches (Suvarna et al. 2024; Njei et al. 2024). However, these therapies may be costly or associated with adverse effects. In contrast, plant‐derived nutraceuticals like quercetin offer a potentially safer, affordable, and complementary strategy. When combined with nanotechnology to overcome bioavailability issues, they may serve either as stand‐alone interventions in early disease or as adjuvants alongside pharmacotherapy. Therefore, Que‐CH nanoparticles represent a translationally attractive alternative within the MASLD therapeutic landscape.

### 4.5 Limitations and Future Directions

Several limitations warrant consideration. First, this study utilized an acute experimental model; long‐term studies are required to assess effects on fibrosis and cirrhosis. Second, pharmacokinetic profiling and tissue biodistribution analyses were not performed. Third, comprehensive toxicity and safety evaluations are needed before clinical translation. Future investigations should also explore dose optimization, combination therapy, and human clinical trials to validate therapeutic efficacy.

## 5 Conclusion

In conclusion, the results of this study confirm that Que‐CH nanoparticles possess significant anti‐inflammatory, anti‐apoptotic, and lipid‐lowering effects, highlighting their potential as a promising therapeutic option for MASLD. These findings support the use of quercetin‐loaded chitosan nanoparticles as an effective strategy for MASLD treatment, providing a novel approach for future research and clinical applications. Nevertheless, further investigations are needed to fully elucidate their mechanisms of action, optimize their formulation, and evaluate their long‐term efficacy in clinical settings. Overall, our results confirm the study hypothesis and underscore the novelty of the quercetin–chitosan nanoformulation, positioning Que‐CH nanoparticles as a promising, multi‐target, and translational therapeutic approach for MASLD.

## Author Contributions

The conception and design of the study were done by “B.M.” Generation collection, assembly, analysis, and interpretation of data were performed by “B.B.”, “M.O.”, “A.T.”, “E.Z.”, “S.N.” and “B.M.” Drafting or revision of the manuscript was done by “B.B.”, “M.O.”, and “B.M.”, and all of the authors approved the final version of the manuscript.

## Funding

This study was supported by Fasa University of Medical Sciences (400286).

## Ethics Statement

IR.FUMS.AEC.1401.002.

## Conflicts of Interest

The authors declare no conflicts of interest.

## Data Availability

The Datasets generated and analyzed during the current study are available from the corresponding author upon reasonable request.
